# Genetic Polymorphism and Natural Selection of Apical Membrane Antigen-1 in *Plasmodium falciparum* Isolates from Vietnam

**DOI:** 10.3390/genes12121903

**Published:** 2021-11-27

**Authors:** Jung-Mi Kang, Hương Giang Lê, Tuấn Cường Võ, Haung Naw, Won Gi Yoo, Woon-Mok Sohn, Nguyen Thi Minh Trinh, Huynh-Hong Quang, Byoung-Kuk Na

**Affiliations:** 1 Department of Parasitology and Tropical Medicine, Institute of Health Sciences, Gyeongsang National University College of Medicine, Jinju 52727, Korea; gjm9951001@hanmail.net (J.-M.K.); gianglee291994@gmail.com (H.G.L.); vtcuong241@gmail.com (T.C.V.); haungnaw23@gmail.com (H.N.); wgyoo@gnu.ac.kr (W.G.Y.); wmsohn@gnu.ac.kr (W.-M.S.); 2 Department of Convergence Medical Science, Gyeongsang National University, Jinju 52727, Korea; 3 Tropical Diseases Clinical and Treatment Research Department, Institute of Malariology, Parasitology and Entomology Quy Nhon, MoH, 611B Nguyen Thai Hoc Street, Quy Nhon 611B, Vietnam; nguyenminhtrinh1983@gmail.com (N.T.M.T.); huynhquangimpe@yahoo.com (H.-H.Q.)

**Keywords:** *Plasmodium falciparum*, apical membrane antigen-1, genetic polymorphism, natural selection, Vietnam

## Abstract

Apical membrane antigen-1 of *Plasmodium falciparum* (PfAMA-1) is a leading malaria vaccine candidate antigen. However, the genetic diversity of *pfama-1* and associated antigenic variation in global *P. falciparum* field isolates are major hurdles to the design of an efficacious vaccine formulated with this antigen. Here, we analyzed the genetic structure and the natural selection of *pfama-1* in the *P. falciparum* population of Vietnam. A total of 37 distinct haplotypes were found in 131 *P. falciparum* Vietnamese isolates. Most amino acid changes detected in Vietnamese *pfama-1* were localized in the ectodomain, domains I, II, and III. Overall patterns of major amino acid changes in Vietnamese *pfama-1* were similar to those of global *pfama-1*, but the frequencies of the amino acid changes slightly differed by country. Novel amino acid changes were also identified in Vietnamese *pfama-1*. Vietnamese *pfama-1* revealed relatively lower genetic diversity than currently analyzed *pfama-1* in other geographical regions, and suggested a distinct genetic differentiation pattern. Evidence for natural selection was detected in Vietnamese *pfama-1*, but it showed purifying selection unlike the global *pfama-1* analyzed so far. Recombination events were also found in Vietnamese *pfama-1*. Major amino acid changes that were commonly identified in global *pfama-1* were mainly localized to predicted B-cell epitopes, RBC-binding sites, and IUR regions. These results provide important information for understanding the genetic nature of the Vietnamese *pfama-1* population, and have significant implications for the design of a vaccine based on PfAMA-1.

## 1. Introduction

Despite remarkable reduction in global mortality and morbidity of malaria in recent years, the disease is still a global public health concern. Approximately 229 million clinical cases of malaria, with an estimated 409,000 deaths, have been reported in 2019 [1]. Development of an efficacious vaccine is highly imperative, considering the huge socio-economic impact of global malaria. However, no effective malaria vaccine is commercially available despite extensive global efforts. The emergence and spread of parasites with antimalarial drug resistance are also a great hindrance for the effective control and elimination of malaria [2].

Apical membrane antigen-1 (AMA-1) of the *Plasmodium* species is a membrane protein consisting of a signal sequence, a cysteine-rich ectodomain, a conserved cytoplasmic region, and a transmembrane region [3]. The ectodomain is further segmented into three distinct domains, domains I, II, and III [3]. This protein is mainly expressed in the electron-dense neck of rhoptries of sporozoites and merozoites, and plays an important function in the invasion of hepatocytes and erythrocytes by contributing attachment of the target cells at the posterior end of *Plasmodium* parasites [4,5,6,7,8]. AMA-1 is a highly immunogenic protein, and evokes a natural immune response in patients infected with either *P. falciparum* or *P. vivax* [9,10,11,12]. Immunization with recombinant AMA-1 elicits antibodies to hinder erythrocyte invasion by the malaria parasite, and confers a protective immune response [11,12]. Therefore, AMA-1 has been recognized as a leading malaria vaccine candidate [13,14]. However, the antibodies induced by AMA-1 recognize either conserved or allele-specific epitopes of AMA-1, resulting in limited protection against distinct alleles [15,16,17]. Despite the less variable genetic diversity of AMA-1 compared to other vaccine candidate antigens, such as circumsporozoite protein (CSP), Duffy-binding protein (DBP), and merozoite surface protein-1 (MSP-1), in the global population, it manifests irrefutable polymorphism [18,19,20,21,22,23,24,25,26,27]. Genetic polymorphism of AMA-1 in global *Plasmodium* field isolates, and the resulting variants in different geographic areas, are major hurdles in the development of a global malaria vaccine based on this antigen. Therefore, it is important to monitor genetic variations in the vaccine candidate antigen in the global *Plasmodium* isolates, since accumulated or newly emerging mutations can change the structure of the antigen, making it difficult to design optimized malaria vaccines.

Malaria was one of the most prominent infectious diseases affecting high mortality in Vietnam until the early 1990s, but the malaria burden in the country has been dramatically reduced over the past few decades [1]. Between 2012 and 2018, the cases and deaths of malaria were reduced by 74% and 95%, respectively, in Vietnam. The National Malaria Control and Elimination Program (NMCEP) of Vietnam has aimed for malaria elimination in the country by 2030 [28]. However, the Central Highlands of Vietnam, which are forests or forest edges, is still a high malaria risk area [29], and *P. falciparum* is a dominant species circulating in the Central Highlands [30,31]. Concerns for the emergence and spread of antimalarial drug-resistant parasites also have increased [32,33,34,35]. In the present study, we analyzed the genetic structure and natural selection of *P. falciparum* AMA-1 (*pfama-1*) in *P. falciparum* isolates from Vietnam to expand our knowledge on genetic variations in the global *pfama-1* applicable to the development of a vaccine targeting PfAMA-1.

## 2. Materials and Methods

### 2.1. Blood Samples and Ethics

**Figure 1:**
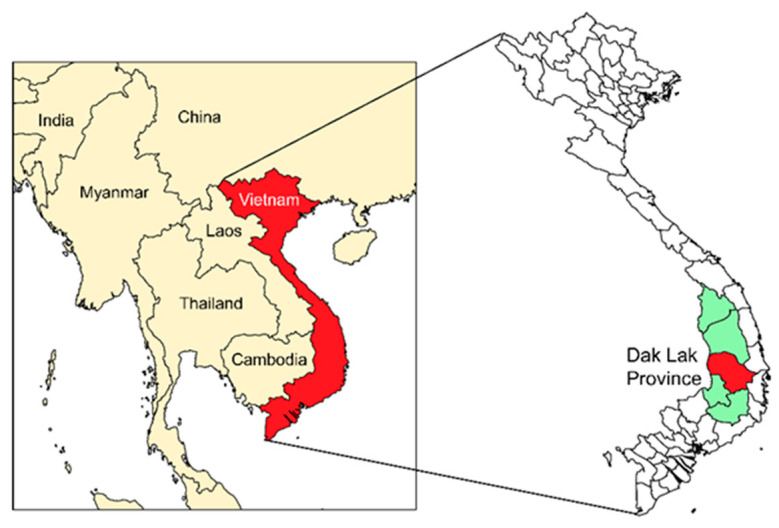
Map of the blood collection sites. Blood samples were collected from *P. falciparum*-infected patients who resided in Dak Lak Province of the Central Highlands, Vietnam in 2019. The Central Highlands region and Dak Lak Province are colored with green and red, respectively.

Blood samples were collected from malaria patients who were infected with *Plasmodium falciparum* in Dak Lak Province, Central Highlands, Vietnam, in 2019 [31] (Figure 1). Malaria infection was initially diagnosed using microscopic examinations for thick and thin blood smears. Finger prick blood samples from the patients were spotted on filter papers (Whatman 3 mm, GE Healthcare, Pittsburg, PA, USA), air-dried, and kept in individual sealed plastic bags at ambient temperature until use. Prior to blood collection, written informed consent was obtained from all the patients. *P. falciparum* infections were further validated by polymerase chain reaction (PCR) targeting the 18S ribosomal RNA (rRNA) gene [36,37]. The study protocols were reviewed and approved by the Ethics Committee of the Ministry of Health, Institute of Malariology, Parasitology, and Entomology (IMPE), Quy Nhon, Vietnam (No. 368/VSR-LSDT).

### 2.2. Amplification and Sequence Analysis of pfama-1

Parasite genomic DNA was isolated from the blood spots using QIAamp DNA Blood Kit (Qiagen, Hilden, Germany) according to the manufacturer’s protocol. Full-length *pfama-1* was amplified from the genomic DNA by nested PCR using primer sets and thermal cycles, as described previously [25]. To minimize nucleotide misincorporation into sequences during amplification steps, Ex Taq DNA polymerase (Takara, Otsu, Japan) with proofreading activity was used in all PCR procedures. Each PCR product was analyzed by electrophoresis on 1% agarose gel, purified from the gel, and cloned into a T&A cloning vector (Real Biotech Corporation, Banqiao City, Taiwan). Each ligation mixture was transformed into *Escherichia coli* DH5α competent cells, and positive clones were selected by colony PCR with the nested PCR primers [25]. Nucleotide sequences of cloned genes were analyzed by DNA sequencing using M13 forward and M13 reverse primers. Sequencing was also performed with two internal primers (5′-CAGGGAAATGTCCAGTATTTGGTA-3′ and 5′-TTCCATCGACCCATAATCCG-3′) to get confidential sequences corresponding to the central region of full-length *pfama-1* [25]. To ensure sequencing accuracy, at least three clones from each isolate were sequenced. Some isolates underwent four- or five-fold sequence coverage to confirm the rare polymorphisms. The nucleotide sequences of Vietnamese *pfama-1* were deposited at GenBank (accession numbers MW938322–MW938452).

### 2.3. Nucleotide Sequence Polymorphism and Neutrality Test

Nucleotide and deduced amino acid sequences of *pfama-1* were analyzed using EditSeq and SeqMan programs in the DNASTAR package (DNASTAR, Madison, WI, USA). Nucleotide sequence polymorphism of Vietnamese *pfama-1* sequences was analyzed. The numbers of segregating sites (S), average number of pair-wise nucleotide differences within a population (*K*), haplotypes (H), haplotype diversity (Hd), and nucleotide diversity (π) were analyzed using DnaSP ver. 5.10.00 [38]. The π value was calculated to estimate step-wise diversity throughout the full-length *pfama-1* based on a sliding window of 100 bp with a step size of 25 bp. Non-synonymous (dN) and synonymous (dS) substitutions were estimated and were compared with Z-test (*p* < 0.05 was considered significant) in the MEGA4 program [39] using the Nei and Gojobori method [40] with Jukes and Cantor correction. Tajima’s D value [41], and Fu and Li’s D and F values [42] were analyzed using DnaSP ver. 5.10.00 to test the neutral theory of evolution [38]. The recombination parameter (R), which included the effective population size and probability of recombination between adjacent nucleotides per generation, and the minimum number of recombination events (Rm) were analyzed with DnaSP ver. 5.10.00 [38]. Linkage disequilibrium (LD) between different polymorphic sites was analyzed based on the R^2^ index using DnaSP ver. 5.10.00 [38].

### 2.4. Population Diversity of pfama-1 among Global P. falciparum Isolates

The genetic diversity of *pfama-1* in global *P. falciparum* isolates was analyzed. The full-length *pfama-1* sequences available in public databases were included: Ghana (*n* = 37, AB715698–AB715734); Myanmar (*n* = 58, KU893276–KU893333); Papua New Guinea (PNG: *n* = 90, AB715870–AB715959); Philippines (*n* = 55, AB715815–AB715869); Solomon Islands (*n* = 50, AB715960–AB716009); Tanzania (*n* = 62, AB715636–AB715679); Thailand (*n* = 80, AB715735–AB715814); and Vanuatu (*n* = 85, AB716010–AB716094). Nucleotide sequence polymorphism and a neutrality test for each population were analyzed using the DnaSP ver. 5.10.00 [38] and MEGA4 [39] programs as described above. Genetic differentiation of parasite populations was calculated based on fixation index (*F_ST_*) to estimate pair-wise DNA sequence diversity between and within populations using DnaSP ver. 5.10.00 [38]. To determine the relationships between global *pfama-1* haplotypes, the haplotype network of 648 *pfama-1* sequences, including 131 from Vietnam and the 517 from Ghana, Myanmar, PNG, Philippines, Solomon Islands, Tanzania, Thailand, and Vanuatu, was constructed using the program PopART version 1.7 (http://popart.otago.ac.nz; accessed on 13 September 2021) with the Median Joining algorithm [43]. To evaluate the association between genetic diversity of *pfama-1* within *P. falciparum* isolates and host immune pressure, genetic diversity in predicted B-cell epitopes, intrinsically unstructured/disordered regions (IUR), and red blood cell (RBC) binding regions in global *pfama-1* were analyzed [25,44,45]. The nucleotide diversity and natural selection of each region were analyzed using DnaSP ver. 5.10.00 [38].

## 3. Results

### 3.1. Sequence Polymorphism of Vietnamese pfama-1

**Figure 2:**
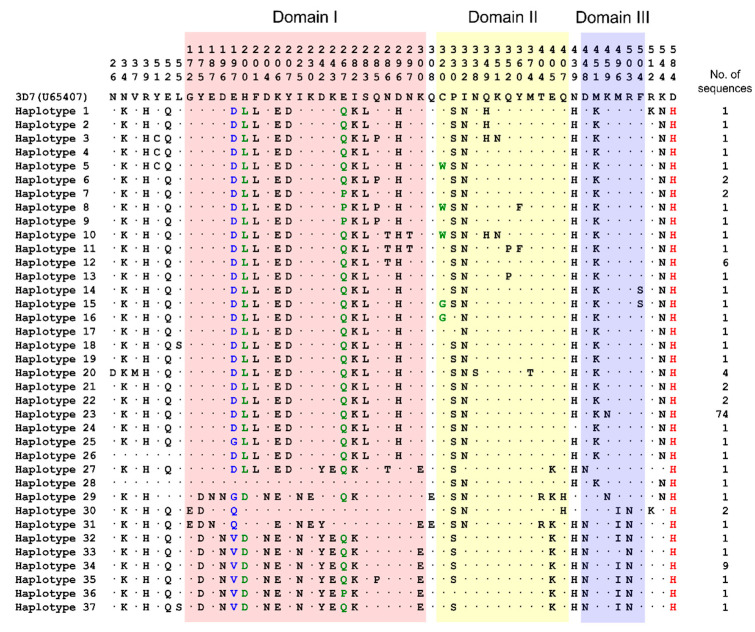
Amino acid sequence polymorphisms in the Vietnamese *pfama-1*. A total of 131 *pfama-1* sequences were obtained from 135 Vietnamese *P. falciparum* isolates. Sequence analysis of the sequences revealed that 64 non-synonymous SNPs were detected in Vietnamese *pfama-1*; resulting amino acid changes at 52 positions compared to *pfama-1* of 3D7 (GenBank Accession No: U65407). Most amino acid changes were identified in domains I, II, and III. Identical amino acid residues with 3D7 sequences were indicated by dots. Based on these amino acid polymorphisms, Vietnamese *pfama-1* classified into 37 distinct haplotypes. These amino acid changes were unevenly scattered in each Vietnamese *pfama-1* haplotype. However, few amino acid changes were commonly identified in Vietnamese *pfama-1* with high frequencies. Conserved amino acid change identified in all Vietnamese *pfama-1* are marked as red. Tri-morphic amino acid changes (H200D/L, E267P/Q, and C320G/W) are presented as green. Penta-morphic amino acid changes (E197D/G/Q/V) are marked as blue. Domains I, II, and III are highlighted as shading with different colors, red, yellow, and blue, respectively.

Nested PCR of *pfama-1* from 135 *P. falciparum* samples from Vietnam resulted in successful amplification of *pfama-1* in 131 samples. No amplicon was detected in four *P. falciparum* isolates. The size of the amplified products was approximately 1.9 kbp, and no size variation was detected between and among the amplified products. Nucleotide sequence analysis of the 131 Vietnamese *pfama-1* sequences based on *pfama-1* from 3D7 reference strain (GenBank accession number: U65407) revealed 116 single nucleotide polymorphisms (SNPs), including 52 synonymous and 64 non-synonymous SNPs. The non-synonymous SNPs induced amino acid substitutions at 52 positions in Vietnamese *pfama-1* sequences, resulting in 37 distinct haplotypes of *pfama-1* in the amino acid levels (Figure 2). These amino acid changes were scattered throughout each haplotype of Vietnamese *pfama-1*, but most of the amino acid changes were detected in domain I (22 positions), domain II (12 positions), and domain III (6 positions). Most amino acid changes were di-morphic (48 positions), but tri-morphic amino acid changes at three positions (H200L/D, E267P/Q, and C320G/W), and a penta-morphic amino acid change at one position (E197D/G/Q/V) were also detected. One amino acid change (D584H) was commonly detected in all Vietnamese *pfama-1* sequences (Figure 2). Of the amino acid changes found in Vietnamese *pfama-1*, amino acid changes at 41 positions were previously identified in *pfama-1* of *P. falciparum* isolates from other geographical areas. However, the other 12 changes at 11 positions (N26D, V37M, Y51C, L55S, C320G/W, N338S, Q352P, Y360F, M374T, K459N, and I504S) were novel and never reported previously in global *pfama-1*, despite their low frequencies, ranging from 1.5 to 3.8%. Haplotype 23 was the most prevalent haplotype, accounting for 56.5% (74/131), followed by haplotype 34 (6.9%; 9/131), haplotype 12 (4.6%; 6/131), and haplotype 20 (3.1%; 4/131). Other haplotypes were detected in only one or two sequences resulting in low frequencies, respectively.

### 3.2. Amino Acid Polymorphisms in Vietnamese pfama-1 Compared with Global pfama-1

**Figure 3:**
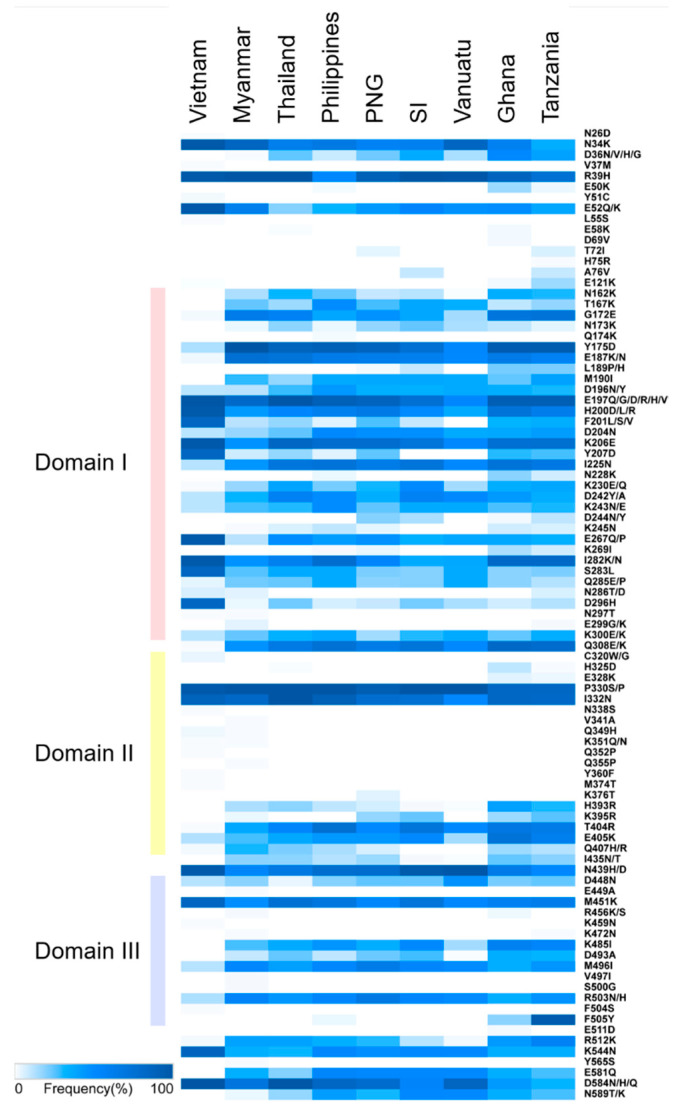
Summary of amino acid polymorphisms of *pfama-1* observed in global *P. falciparum* isolates. Positions and frequencies of amino acid changes detected in global *pfama-1* were compared. Each domain is highligthed by distinct colors: domain I (red); domain II (yellow); and domain III (blue). Overall patterns of amino acid changes detected in global *pfama-1* were similar, but frequencies of each amino acid change differed by country. Some amino acid changes were characterized by country or continent. PNG, Papua New Guinea; SI, Solomon Islands. Heatmap was generated using Morpheus (https://software.broadinstitute.org/morpheus/; accessed on 16 November 2021). The global patterns except Vietnamese *pfama-1* have been analyzed previously [25], and the previous data were applied to analyze overall patterns.

Vietnamese *pfama-1* shared similar, but not identical, patterns of amino acid polymorphism with global *pfama-1*. Amino acid changes at 91 positions were identified in global *pfama-1*. Most amino acid changes were detected in domains I (33 positions), II (19 positions), and III (16 positions), but also at 5′-terminal (5′-T) and 3′-terminal (3′-T) regions (Figure 3). Although global *pfama-1* showed similar patterns of amino acid changes, the frequency of each amino acid change differed by country. Amino acid changes at 11 positions in Vietnamese *pfama-1* showed higher frequencies than *pfama-1* from other countries: N34K (Vietnam, 98.5%; others, 35.5–86.2%); E52Q/K (Vietnam, 97.7%; others, 20.0–62.1%); H200D/L/R (Vietnam, 96.9%; others, 36.5–73.0%); F201L/S/V (Vietnam, 85.5%; others, 0.0–35.5%); K206E (Vietnam, 97.7%; others, 51.8–83.8%); Y207D (Vietnam, 85.5%; others, 0.0–29.7%); E267Q/P (Vietnam, 96.9%; others, 12.1–48.8%); I282K/N (Vietnam 96.9%; others 36.0–82.3%); S283L (Vietnam, 84.7%; others, 20.0–36.5%); D296H (Vietnam, 84.7%; others, 3.4–22.5%); and K544N (Vietnam, 86.3%; others, 31.3–52.7%). Amino acid changes at 12 positions were detected with lower frequencies in Vietnamese *pfama-1* compared with *pfama-1* sequences from other countries: G172E (Vietnam, 2.3%; others, 15.3–72.6%); Y175D (Vietnam, 13.7%; others, 52.9–100%); E187K/N (Vietnam, 3.1%; others, 50.6–75.9%); I225N (Vietnam, 12.2%; others, 51.8–73.0%); K230E/Q (Vietnam, 1.5%; others, 15.3–38.8%); D242Y/A (Vietnam, 11.5%; others, 32.8–58.8%); K243N/E (Vietnam, 11.5%; others, 24.4–49.1%); Q285E/P (Vietnam 4.6%; others 12.9–36.5%); Q308E/K (Vietnam, 1.5%; others, 46.6–81.1%); T404R (Vietnam, 1.5%; others, 37.9–76.4%); M496I (Vietnam, 12.2%; others, 35.1–64.4%); and R503N/H (Vietnam, 13.7%; others, 35.1–66.7%). Amino acid changes at 11 positions (D36N/V/H/G, N162K, T167K, N173K, M190I, H393R, I435N/T, K483I, D493A, E581Q, and N589T/K), which were commonly identified in other countries, were not detected in Vietnamese *pfama-1*. Meanwhile, 12 amino acid changes at 11 positions (N26D, V37M, Y51C, L55S, C320G/W, N338S, Q352P, Y360F, M374T, K459N, and F504S) were unique to Vietnamese *pfama-1*, and not detected in global *pfama-1* [25]. Substitution rates of amino acid changes at five positions (R39H, E197Q/G/D/R/H/V, P330S/P, I332N, and N439H/D) were relatively high (at least up to 50%) in all global *pfama-1* analyzed.

### 3.3. Nucleotide Diversity and Natural Selection of Vietnamese pfama-1

**Figure 4:**
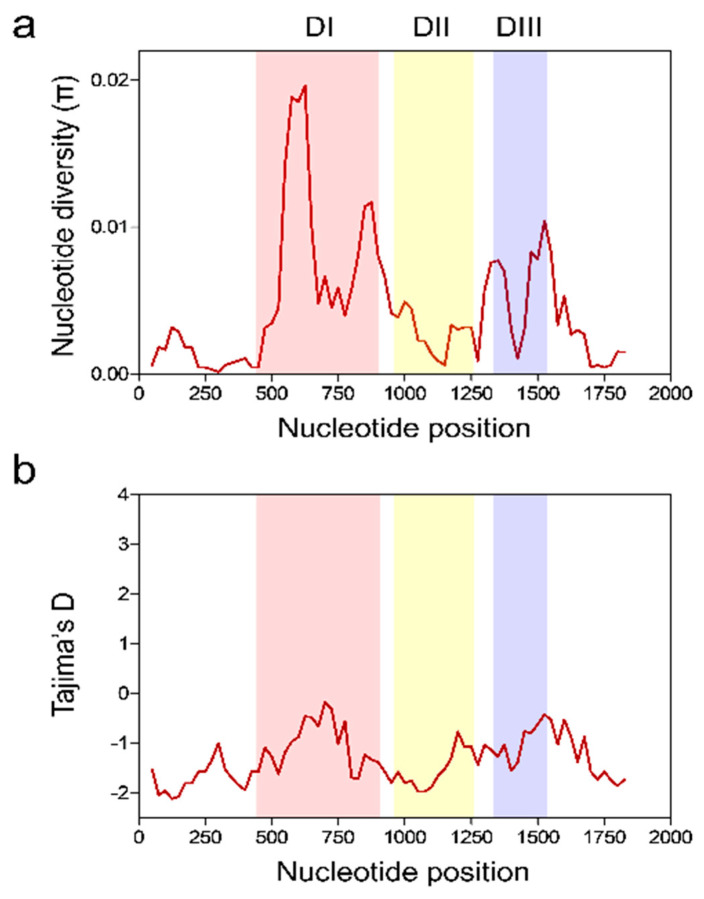
Nucleotide diversity and natural selection of Vietnamese *pfama-1*. (**a**) Nucleotide diversity. Sliding window plot showed nucleotide diversity (π) values across Vietnamese *pfama-1* sequences. A window size of 100 bp and a step size of 25 bp were applied. (**b**) Natural selection. Sliding window plot of Tajima’s D was analyzed for Vietnamese *pfama-1*. A window size of 100 and a step size of 25 were applied. DI, domain I; DII, domain II; DIII, domain III.

The nucleotide difference (*K*) of full-length *pfama-1* of Vietnam isolates was 8.062 (Table 1). The highest *K* was identified at domain I (*K* = 4.198), whereas the lowest was detected at 5′-T (*K* = 0.485). Haplotype diversity (Hd) for 131 full-length Vietnamese *pfama-1* was 0.837 ± 0.034. This value was higher at domain I (0.575 ± 0.052) than at 5′-T (0.358 ± 0.055), domains II (0.465 ± 0.053), domain III (0.455 ± 0.053), and 3′-T (0.432 ± 0.053). The nucleotide diversity (π) of each fragment was 0.0011 ± 0.0002 (5′-T), 0.0091 ± 0.0014 (domain I), 0.0031 ± 0.0004 (domain II), 0.0076 ± 0.0012 (domain III), and 0.0023 ± 0.0003 (3′-T), respectively (Table 1). Analysis of π across the Vietnamese *pfama-1* also revealed two major peaks at domains I and III (Figure 4a). To determine whether natural selection affected the diversity of Vietnamese *pfama-1*, the dN − dS value was calculated using Nei and Gojobori’s method [40]. The dN − dS value for full-length Vietnamese *pfama-1* was 0.0009, implying that no or mild natural selection might have acted in the Vietnamese *pfama-1*. However, slightly higher dN − dS values were identified at domain I (0.0084) and domain II (0.0020), implying the impact of mild natural selection. Tajima’s D value for full-length Vietnamese *pfama-1* was −2.0046 (*p* < 0.05) (Table 1). When Tajima’s D value was analyzed for each fragment, all fragments showed negative, suggesting selective sweep or purifying selection. A sliding window plot of Tajima’s D values across the Vietnamese *pfama-1* also showed that the values were below zero in all the domains (Figure 4b).

### 3.4. Nucleotide Diversity and Natural Selection of Global pfama-1

**Figure 5:**
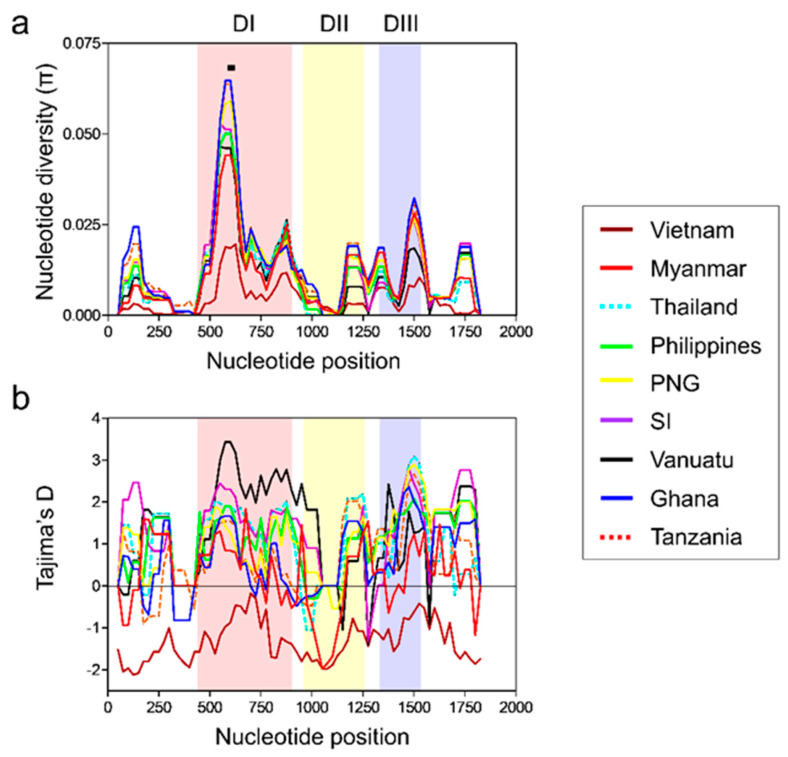
Nucleotide diversity and natural selection of global *pfama-1*. (**a**) Nucleotide diversity. Sliding window plot showed nucleotide diversity (π) values across global *pfama-1* sequences. A window size of 100 bp and a step size of 25 bp were applied. The cluster 1 of loop I (C1-L) region, which is located on the π peak of domain I, is marked with a black line. (**b**) Natural selection. Sliding window of Tajima’s D was analyzed for global *pfama-1*. A window size of 100 and a step size of 25 were applied. The patterns of π and Tajima’s D of *pfama-1* from each county are represented with different colors. DI, domain I; DII, domain II; DIII, domain III. PNG, Papua New Guinea; SI, Solomon Islands. The global patterns except Vietnamese *pfama-1* have been analyzed previously [25], and the previous data were applied to analyze overall patterns.

The Vietnamese *pfama-1* showed a substantially lower *K* value compared with the global *pfama-1* including the Southeast Asian countries, Myanmar and Thailand (Table 2). The *K* values of African *pfama-1* were higher than those of Asian and Pacific *pfama-1*. The Hd values of African *pfama-1* were also higher than those of *pfama-1* derived from other geographic areas. In contrast to other global *pfama-1*, the Vietnamese *pfama-1* showed substantially low-level selective pressure, with a dN/dS ratio of 1.250, suggesting minimal positive selection. The π value of *pfama-1* from each country differed by country, whereas African *pfama-1* showed higher π values than those of Asian and Pacific *pfama-1* (Table 2). Vietnamese *pfama-1* showed the lowest π value among the global *pfama-1* analyzed. To determine the occurrence and significance of any deviation from neutral evolution, Tajima’s D, and Fu and Li’s D and F values were calculated. All *pfama-1* sequences except Vietnamese *pfama-1* showed positive Tajima’s D values, indicating balancing selection in global *pfama-1* (Table 2). Both Fu and Li’s D and F values also suggested evidence for balancing selection acting primarily on the global *pfama-1*. However, interestingly, the Vietnamese *pfama-1* revealed a negative Tajima’s D value, implying selective sweep or purifying selection. A sliding window plot of π revealed that global *pfama-1* shared highly similar patterns of π across the sequences (Figure 5a). The highest peak of π was commonly identified at domain I of all global isolates. The cluster 1 of the loop I (C1-L) region was located on the π peak of domain I. A sliding window plot of Tajima’s D also revealed that global *pfama-1* exhibited a similar pattern of Tajima’s D across the gene, with positive values at domains I and III, despite a few differences among global *pfama-1* (Figure 5b). However, the Vietnamese *pfama-1* showed a different pattern of Tajima’ D, with negative values across the gene, even though Vietnam *pfama-1* showed comparable patterns of two peaks at domains I and III consistent with global *pfama-1*.

### 3.5. Recombination and Linkage Disequilibrium

**Figure 6:**
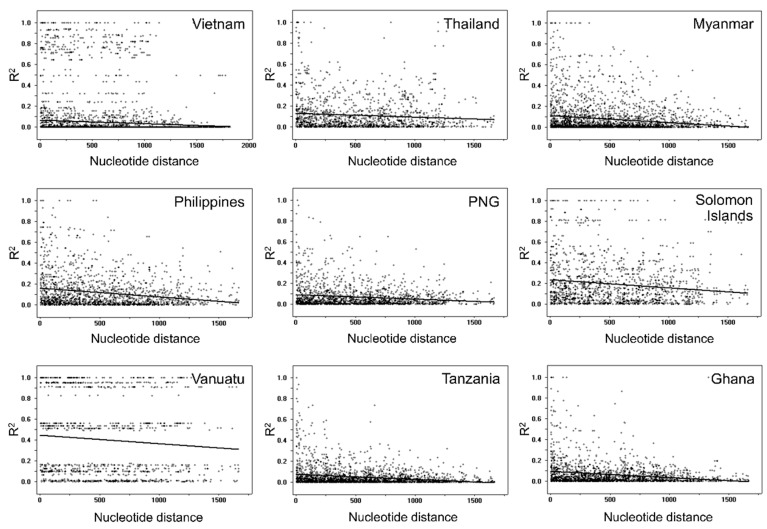
Recombination event in global *pfama-1*. Linkage disequilibrium (LD) plot suggested non-random associations between nucleotide variations in *pfama-1* at different polymorphic sites. R^2^ values were plotted against nucleotide distance using a two-tailed Fisher’s exact test for statistical significance. PNG, Papua New Guinea. The global patterns except Vietnamese *pfama-1* have been analyzed previously [25], and the previous data were applied to analyze overall patterns.

The estimated minimum number of recombination events between adjacent polymorphic sites (Rm) for Vietnamese *pfama-1* was 20 (Table 3). The predicted location of plausible recombination sites were domains I and III, suggesting that meiotic recombination between the sites may have contributed to the genetic diversity of Vietnamese *pfama-1*. Possible recombination events were also predicted in global *pfama-1*. The highest R values were detected in African *pfama-1* (Ghana and Tanzania), whereas Asian and Pacific *pfama-1*, except PNG, showed relatively lower R values (Table 3). The LD index (R^2^) for global *pfama-1* also declined with increasing distance across the gene, suggesting the role of intragenic recombination in the genetic diversity of global *pfama-1* (Figure 6).

### 3.6. Haplotype Network Analysis

**Figure 7:**
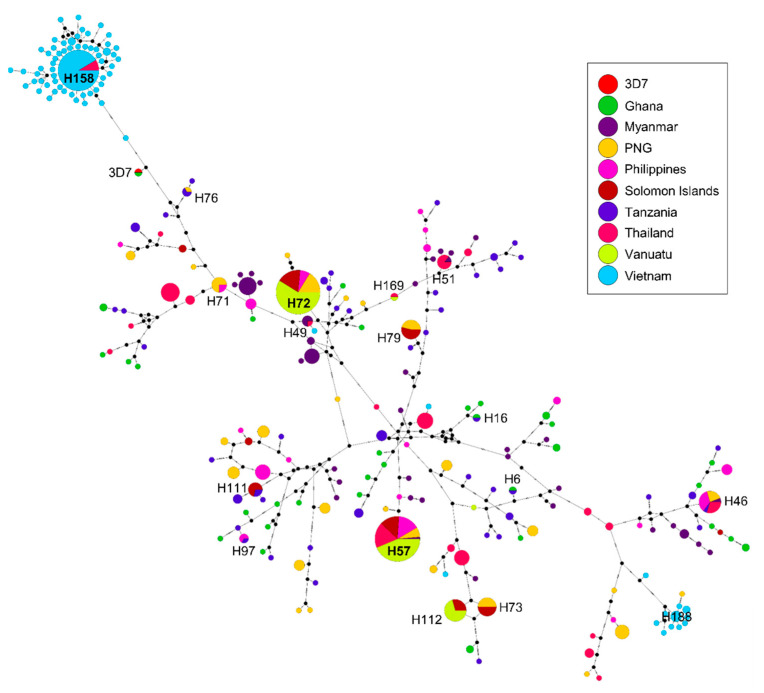
Network analysis of global *pfama-1* haplotypes. Haplotype network was constructed using the program PopART version 1.7 with the Median Joining algorithm. A total of 648 global *pfama-1* sequences were analyzed. The size of each node indicates the frequency of a particular haplotype. The lengths of the lines connecting the nodes are in proportion to the number of base pair substitutions separating the haplotypes. Color of each node indicates each country.

In order to analyze the relationships between and among the global *pfama-1*, a haplotype network was constructed. A dense network with 245 individual haplotypes with complicated relationships was established using 648 global *pfama-1* sequences (Figure 7). Haplotype 57 was a predominant haplotype shared by isolates from six Asian and Pacific countries, including Myanmar, Thailand, Philippines, PNG, Solomon Islands, and Vanuatu. Haplotype 72 (H72) was also a major haplotype, with a prevalence of 10.5%, and shared by Pacific populations. Four haplotypes (H71, H73, H79, and H112) were admixed with Pacific populations. H6 and H16 comprised solely African populations. Five haplotypes (H46, H51, H76, H97, and H111) were shared by African populations and Asian or Pacific populations. Interestingly, most Vietnamese *pfama-1* haplotypes did not cluster with Asian and Pacific populations, and instead clustered into two separated haplotype groups, which branched from H158 and H188.

### 3.7. Nucleotide Differentiation among Global pfama-1

To further analyze the genetic differentiation and gene flow in global *pfama-1*, *F_ST_* values were analyzed (Table 4). The *F_ST_* values between different geographical *pfama-1* populations ranged from 0.00019 (between Ghana and Tanzania) to 0.42009 (between Vietnam and Solomon Islands). Interestingly, the Vietnamese *pfama-1* exhibited large genetic differentiation from other global *pfama-1*.

### 3.8. Association between Natural Selection and Host Immune Pressure

**Figure 8:**
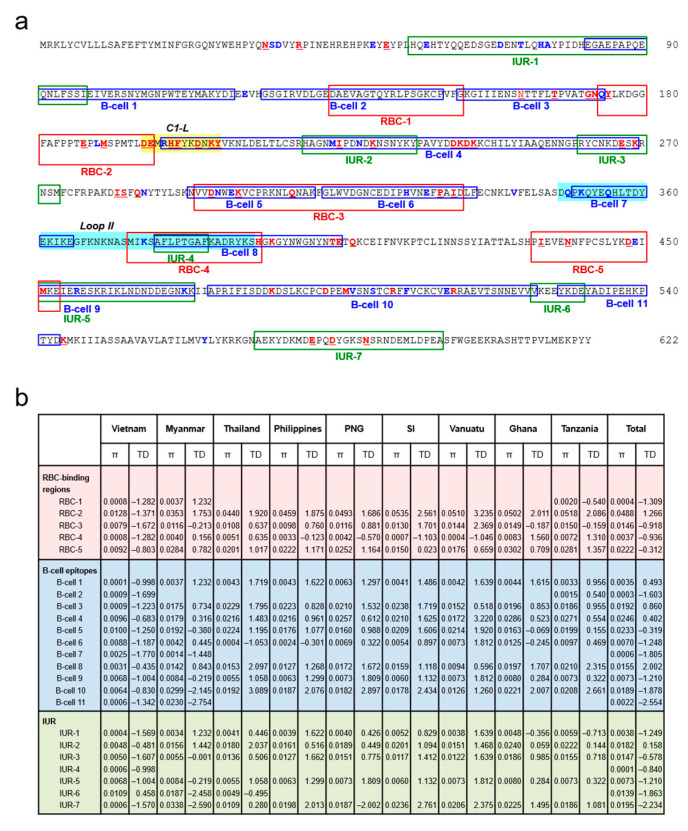
Association between natural selection and host immune pressure. (**a**) Positions of amino acid changes detected in global *pfama-1* and predicted B-cell epitopes, RBC-binding regions, and IUR regions. Predicted B-cell epitopes, RBC-binding regions [44], and IUR regions [45] are represented by blue boxes, red boxes, and green boxes, respectively. Polymorphic amino acid residues commonly detected in global *pfama-1* are marked as bold red. The less commonly detected amino acid changes are shown as bold blue. The C1-L (cluster 1 of loop I, aa: 196–207) in DI, and the loop II (aa: 348–392) in DII are marked with yellow and sky-blue squares, respectively. (**b**) Nucleotide diversity and natural selection analysis. Nucleotide diversity (π) and Tajima’s D (TD) values for each B-cell epitope region, RBC-binding region, and IUR region in global *pfama-1* were analyzed using DnaSP program. PNG, Papua New Guinea; SI, Solomon Islands. The global patterns except Vietnamese *pfama-1* have been analyzed previously [25], and the previous data were applied to analyze overall patterns.

To evaluate selective pressure of host immunity on *pfama-1*, the genetic polymorphisms in the predicted B-cell epitopes, RBC-binding sites, and IUR regions of *pfama-1* were analyzed. Most major amino acid changes were detected in the predicted B-cell epitopes, RBC-binding sites, or IUR regions of *pfama-1* (Figure 8a). Of 91 amino acid changes identified in global *pfama-1* compared with the 3D7 sequence (GenBank accession No.: U65407), 72 were found at the predicted B-cell epitopes, RBC-binding sites, or IUR regions. Among 51 amino acid changes detected commonly in global *pfama-1*, 42 were located in the predicted B-cell epitopes, RBC-binding sites, or IUR regions. Twenty-nine less common amino acid changes in global *pfama-1* were also detected in the predicted B-cell epitopes, RBC-binding sites, or IUR regions. Eight of eleven predicted B-cell epitopes were polymorphic. Particularly, B-cell epitopes 3, 4, 5, 8, and 10 had major polymorphic amino acid residues with high levels of *π* (Figure 8b). Tajima’s D values for the predicted B-cell epitopes 3, 4, and 8 were positive, whereas the values for the predicted B-cell epitopes 5 and 10 were negative (Figure 8b). Amino acid changes commonly identified in global *pfama-1* were mainly detected at the C1-L region, which is localized near the hydrophobic pocket of PfAMA-1 [46], and corresponded to the π peak in domain I. A few less frequently observed amino acid changes in global *pfama-1* were detected in the loop II region.

## 4. Discussion

In the present study, we analyzed the genetic polymorphisms and natural selection of Vietnamese *pfama-1* in order to understand the genetic nature of Vietnamese *pfama-1*. Diverse amino acid changes resulted by SNPs were detected in Vietnamese *pfama-1*, similar to global *pfama-1* reported from other countries [25,45,47,48,49,50]. The 51 major amino acid changes that are commonly detected in global *pfama-1* were also observed in Vietnamese *pfama-1*, but their patterns and frequencies differed with global populations. The majority of the common amino acid changes were localized in domains I, II, and III, supporting that these domains are major regions contributing to *pfama-1* polymorphism [25]. Some amino acid changes were unique to *pfama-1* of specific countries or continents. In particular, Vietnamese *pfama-1* showed different patterns of amino acid changes from global *pfama-1*. Twelve amino acid changes at 11 positions (N26D, V37M, Y51C, L55S, C320G/W, N338S, Q352P, Y360F, M374T, K459N, and I504S) were unique to Vietnamese *pfama-1*, but not detected in global *pfama-1*. By contrast, 11 amino acid changes (D36N/V/H/G, N162K, T167K, N173K, M190I, H393R, I435N/T, K485I, D493A, E581Q, and N589T/K), which were commonly identified in global *pfama-1* with varying frequencies, were not detected in Vietnamese *pfama-1*. Haplotype network analysis also revealed that Vietnamese *pfama-1* formed distinct clusters, which were clearly distinguished from other global populations, including *pfama-1* from the neighboring Southeast Asian countries of Myanmar and Thailand. These findings suggest that the Vietnamese *pfama-1* exhibited distinct patterns of polymorphism and genetic differentiation compared with other global isolates. The *F_ST_* value, a measure of population substructure based on an analysis of the overall genetic differentiation among populations [51], also suggested genetic differentiation of Vietnamese *pfama-1* from other global populations. Due to the limitations of currently available full-length *pfama-1* sequences from diverse geographical origins, the implications of substantial differentiation of Vietnamese *pfama-1* from global *pfama-1* is currently unclear. Further analysis of genetic polymorphisms of *pfama-1* in a larger number of global *P. falciparum* populations is necessary to appreciate the polymorphic nature and evolutionary linkage of global *pfama-1*.

The π value of global *pfama-1* differed depending on the origin of the isolates. The nucleotide diversity in Vietnam *pfama-1* (π = 0.0043) was relatively lower than in isolates from different geographical areas. Lower malaria transmission in restricted areas of Vietnam compared with other endemic countries may contribute to the low-level genetic diversity in Vietnamese *pfama-1*. However, it is also necessary to consider the global *pfama-1* sequences analyzed in this study were obtained from parasites collected at different time points in each country, which do not reflect the π value of the *pfama-1* populations in the same period. In fact, the genetic structure of *Plasmodium* populations in endemic areas or countries changes dynamically over time due to various factors [52,53,54,55,56]. Different sizes of isolates from each country may also affect the π value of *pfama-1* in each country. Therefore, a systematic analysis of the global *P. falciparum* isolates collected in the same period, especially current, may be necessary to delineate the genetic variations and evolutionary trends of global *pfama-1*. Although the π value of global *pfama-1* differed by country, synchronized patterns of π across *pfama-1* were recognized in global *pfama-1*, including Vietnamese *pfama-1*. A sliding window plot suggested that the nucleotide diversity was unevenly distributed across *pfama-1*, and high levels of π were similarly observed in domains I and III of global *pfama-1*, supporting that the domains are the main regions contributing to the genetic heterogeneity of *pfama-1*.

Natural selection probably affected by the host–immune response and recombination between genetically distinct alleles during meiotic replication in the mosquito midgut have been understood as the two main mechanisms underlying *pfama-1* genetic diversity [25,57,58]. Interestingly, the Vietnamese *pfama-1* showed different patterns of natural selection compared with *pfama-1* from other countries. In contrasts to global *pfama-1*, which showed positive values of Tajima’s D, the Vietnamese *pfama-1* revealed a negative Tajima’s D value, implying purifying selection. The dN/dS ratios of global *pfama-1* were relatively high in all isolates, but the value was much lower in Vietnamese *pfama-1*, suggesting the absence of strong positive selection. These findings suggested that Vietnamese *pfama-1* underwent different natural selection compared with *pfama-1* from other countries, probably due to selective sweep or a bottleneck effect. However, regardless of these differences, the sliding window plot revealed that domains I and III exhibited high values of π and Tajima’s D across global *pfama-1*, implying that these regions represent possible dominant targets for natural selection by host immune response. Indeed, most common amino acid changes identified in global *pfama-1* were mainly scattered in domains I and III, corresponding to B-cell epitopes 3, 4, 5, 9, and 10. The C1-L cluster, which is located near the hydrophobic pocket of domain I in PfAMA-1 [46], is known to affect the binding capacity of inhibitory antibodies, and thereby mediates escape from PfAMA-1 antibodies induced by *P. falciparum* infection or vaccine trials [16,45,59,60,61]. The clusters of amino acid polymorphisms including tri- and hepta-morphic changes scattered in the C1-L region of global *pfama-1* suggest strong natural selection, which further contributes to host immune escape [17,61,62]. Meanwhile, loop II, the target of the 4G2 inhibitory antibody [63], was highly conserved in global *pfama-1*, suggesting that this region might be a vaccine candidate based on PfAMA-1 [25]. Meiotic recombination is also one of the main forces driving allelic diversity of *pfama-1* [25,50,57]. Substantial levels of recombination events in *pfama-1* derived from other geographical isolates have been reported [25]. Although the Vietnamese *pfama-1* revealed a lower level of genetic diversity than the other global *pfama-1*, a comparable level of recombination events and decline of the LD index R^2^ were also detected in Vietnamese *pfama-1*. These findings suggest that interallelic recombination is another force generating genetic diversity of the Vietnamese *pfama-1*, and consistent with findings from other geographical areas.

## 5. Conclusions

Overall patterns of nucleotide diversity and major amino acid changes in Vietnamese *pfama-1* were similar to those seen in global *pfama-1*. However, the Vietnamese *pfama-1* revealed relatively lower genetic diversity than *pfama-1* populations from other geographical regions, and showed a distinct genetic differentiation profile. Although evidence for natural selection and recombination, which may contribute to the generation and maintenance of genetic diversity of *pfama-1*, were also found in Vietnamese *pfama-1,* it revealed a distinct trend of natural selection compared with other global *pfama-1*. These results have significant implications for understanding the genetic nature of the Vietnamese *P. falciparum* population, warranting continuous monitoring of the genetic diversity of global *pfama-1* to elucidate the polymorphic nature and evolutionary aspects of *pfama-1*, and design an effective vaccine targeting *P. falciparum* populations globally.

## Figures and Tables

**Table 1:** Estimates of DNA sequence polymorphism, and tests of neutrality at *pfama-1* among *P. falciparum* Vietnam isolates.

Fragment	Segregating Sites (S)	Singleton Variable Sites	Parsimony Informative Sites	Total No. of Mutations	*K*	H	Hd ± SD	π ± SD	dN−dS	Tajima’s D
5′-Terminal	21	12	9	21	0.485	20	0.358 ± 0.055	0.0011 ± 0.0002	−0.0018	−2.4673 (*p* < 0.01)
Domain I	41	12	29	42	4.198	30	0.575 ± 0.052	0.0091 ± 0.0014	0.0084	−1.3889 (*p* > 0.1)
Domain II	17	4	13	18	0.915	21	0.465 ± 0.053	0.0031 ± 0.0004	0.0020	−1.9940 (*p* < 0.05)
Domain III	14	5	9	14	1.526	16	0.455 ± 0.053	0.0076 ± 0.0012	−0.0003	−1.0724 (*p* > 0.1)
3′-Terminal	14	9	5	14	0.772	17	0.432 ± 0.053	0.0023 ± 0.0003	−0.0052	−1.8470 (*p* < 0.05)
Full	114	47	67	116	8.062	73	0.837 ± 0.034	0.0043 ± 0.0006	0.0009	−2.0046 (*p* < 0.05)

S, positions which show differences (polymorphisms) between related genes; singleton variable sites, sites contain at least two types of nucleotides and occur multiple times; parsimony informative sites, sites contain at least two types of nucleotides but only two of them occur with a minimum frequency of two; *K*, average number of pair-wise nucleotide differences; H, number of haplotypes; Hd, haplotype diversity; π, observed average pair-wise nucleotide diversity; dN, rate of non-synonymous mutations; dS, rate of synonymous mutations.

**Table 2:** Estimates of DNA sequence polymorphism and tests of neutrality at *pfama-1* among global *P. falciparum.*

Isolates	Segregating Sites (S)	Singleton Variable Sites	Parsimony Informative Sites	Total No. of Mutations	*K*	H	Hd ± SD	π ± SD	dN/dS	Tajima’s D	Fu and Li’s D	Fu and Li’s F
Vietnam (*n* = 131)	114	47	67	116	8.062	73	0.837 ± 0.034	0.0043 ± 0.0006	1.250	−2.0046 (*p* < 0.05)	−3.1913 (*p* < 0.05)	−3.1959 (*p* < 0.02)
Myanmar ^#^ (*n* = 58)	78	16	62	79	19.886	37	0.948 ± 0.019	0.0106 ± 0.0005	6.524	0.5719 (*p* > 0.1)	0.1677 (*p* > 0.1)	0.3851 (*p* > 0.1)
Thailand ^#^ (*n* = 80)	58	5	53	61	20.621	21	0.920 ± 0.013	0.0110 ± 0.0002	6.698	2.2222 (*p* < 0.05)	0.9572 (*p* > 0.1)	1.7516 (*p* < 0.05)
Philippines ^#^ (*n* = 55)	61	3	58	62	21.232	19	0.916 ± 0.019	0.0114 ± 0.0003	7.200	1.9545 (0.1 > *p* > 0.05)	1.6123 (*p* < 0.05)	2.07960 (*p* < 0.02)
PNG ^#^ (*n* = 90)	68	2	66	75	22.923	28	0.954 ± 0.007	0.0123 ± 0.0002	7.497	1.8082 (0.1 > *p* > 0.05)	1.6759 (*p* < 0.05)	2.0760 (*p* < 0.02)
Solomon Islands ^#^ (*n* = 50)	56	3	53	57	22.044	9	0.862 ± 0.021	0.0118 ± 0.0003	7.023	2.5455 (*p* < 0.05)	1.5447 (*p* < 0.05)	2.2804 (*p* < 0.02)
Vanuatu ^#^ (*n* = 85)	50	6	44	50	18.905	5	0.633 ± 0.028	0.0101 ± 0.0003	6.471	2.8973 (*p* < 0.01)	0.8642 (*p* > 0.1)	2.0039 (*p* < 0.05)
Ghana ^#^ (*n* = 37)	75	7	68	85	26.261	32	0.992 ± 0.008	0.0141 ± 0.0003	7.423	1.0674 (*p* > 0.1)	1.2179 (*p* > 0.1)	1.38500 (*p* > 0.1)
Tanzania ^#^ (*n* = 62)	81	8	73	89	25.444	47	0.989 ± 0.005	0.0136 ± 0.0002	8.135	1.1819 (*p* > 0.1)	1.1635 (*p* > 0.1)	1.4020 (*p* > 0.1)

*K*, average number of pair-wise nucleotide differences; H, number of haplotypes; Hd, haplotype diversity; π, observed average pair-wise nucleotide diversity; dN, rate of non-synonymous mutations; dS, rate of synonymous mutations. ^#^ Cited from [25].

**Table 3:** Comparison of recombination events between global *pfama-1.*

	*n*	Ra	Rb	Rm
Vietnam	131	0.0000	0.001	20
Myanmar ^#^	78	0.0141	26.3	24
Thailand ^#^	38	0.0243	45.3	20
Philippines ^#^	61	0.0218	40.8	17
PNG ^#^	68	0.0500	93.4	28
Solomon Islands ^#^	56	0.0120	22.5	14
Vanuatu ^#^	50	0.0010	1.8	9
Ghana ^#^	75	0.0921	172	27
Tanzania ^#^	81	0.0915	171	28

The R and Rm were estimated excluding the sites containing alignment gaps or those segregating for three nucleotides. The R was computed using R = 4Nr, where N is the population size, and r is the recombination rate per sequence (per gene). *n*, number of sequences analyzed; Ra, recombination parameter between adjacent sites; Rb, recombination parameter for entire gene; Rm, minimum number of recombination events between adjacent sites. ^#^ Cited from [25].

**Table 4:** Pair-wise *F_ST_* estimates for global *pfama-1.*

	Vietnam	Myanmar #	Thailand #	Philippines #	PNG #	SI #	Vanuatu #	Ghana #	Tanzania #
Vietnam		11.56767	12.82408	11.95679	14.11411	11.92480	12.32934	12.07054	13.64608
Myanmar ^#^	0.40780		20.15798	20.35295	21.58878	20.68805	19.15425	22.14497	20.15798
Thailand ^#^	0.37881	0.06073		20.87018	21.83959	21.16846	19.73726	22.40481	22.72679
Philippines ^#^	0.41382	0.08390	0.03818		22.28146	21.61888	19.81950	23.25483	23.46398
PNG ^#^	0.37343	0.05281	0.03818	0.02319		22.60884	20.97135	23.89528	25.95093
SI ^#^	0.42009	0.09605	0.06636	0.03529	0.03529		20.06783	23.83759	23.92600
Vanuatu ^#^	0.40551	0.09740	0.10535	0.06689	0.06689	0.04933		21.13622	21.66300
Ghana ^#^	0.37955	0.07802	0.04140	0.04167	0.03640	0.04953	0.12425		25.74924
Tanzania ^#^	0.39934	0.08139	0.05865	0.05787	0.03662	0.05782	0.14141	0.00019	

*F_ST_* values are represented in the lower left quadrant, and average number of pair-wise nucleotide differences between populations (*K*) are shown in the upper right quadrant. *F_ST_*, a measure of genetic differentiation between populations (range from 0 to +1). PNG, Papua New Guinea; SI, Solomon Islands. ^#^ Cited from [25].

## Data Availability

The data supporting the conclusions of this article are provided within the article. The original datasets analyzed in this study are available from the corresponding author upon request. The nucleotide sequences reported in this study have been deposited in the GenBank database under the accession numbers MW938322–MW938452.
